# Biomedical and Clinical Importance of Mussel-Inspired Polymers and Materials

**DOI:** 10.3390/md13116792

**Published:** 2015-11-11

**Authors:** Nagendra Kumar Kaushik, Neha Kaushik, Sunil Pardeshi, Jai Gopal Sharma, Seung Hyun Lee, Eun Ha Choi

**Affiliations:** 1 Plasma Bioscience Research Center, Kwangwoon University, Seoul 139701, Korea; E-Mails: neha.bioplasma@gmail.com (N.K.); sunil@datacops.org (S.P.); 2 Department of Biotechnology, Delhi Technological University, Delhi 110042, India; E-Mail: sharmajaigopal@gmail.com; 3 Graduate School of Information Contents, Kwangwoon University, Seoul 139701, Korea; E-Mail: shlee@kw.ac.kr

**Keywords:** mussels, mussel-inspired biomedical applications, medical adhesive, wound healing, anti-proliferative, anti-inflammatory, stem cell differentiation, surface coating, nano-constructs

## Abstract

The substance secreted by mussels, also known as nature’s glue, is a type of liquid protein that hardens rapidly into a solid water-resistant adhesive material. While in seawater or saline conditions, mussels can adhere to all types of surfaces, sustaining its bonds via mussel adhesive proteins (MAPs), a group of proteins containing 3,4-dihydroxyphenylalanine (DOPA) and catecholic amino acid. Several aspects of this adhesion process have inspired the development of various types of synthetic materials for biomedical applications. Further, there is an urgent need to utilize biologically inspired strategies to develop new biocompatible materials for medical applications. Consequently, many researchers have recently reported bio-inspired techniques and materials that show results similar to or better than those shown by MAPs for a range of medical applications. However, the susceptibility to oxidation of 3,4-dihydroxyphenylalanine poses major challenges with regard to the practical translation of mussel adhesion. In this review, various strategies are discussed to provide an option for DOPA/metal ion chelation and to compensate for the limitations imposed by facile 3,4-dihydroxyphenylalanine autoxidation. We discuss the anti-proliferative, anti-inflammatory, anti-microbial activity, and adhesive behaviors of mussel bio-products and mussel-inspired materials (MIMs) that make them attractive for synthetic adaptation. The development of biologically inspired adhesive interfaces, bioactive mussel products, MIMs, and arising areas of research leading to biomedical applications are considered in this review.

## 1. Introduction

**Figure 1:**
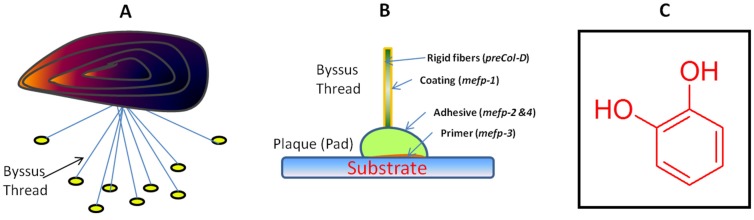
Properties of mussel adhesion: (**A**) biological adhesion; (**B**) bio-inspired adhesion on a substrate; and (**C**) the catechol chemical moiety involved in adhesion.

Mussel adhesion is a natural process which involves the secretion of a type of protein glue that hardens rapidly into a solid and turns into a water-resistant adhesive [1]. Many features of this process have inspired the development of synthetic materials for medical applications. The mussel secretes sticky glue that allows it to stick to rocks and solid surfaces. The important factor to its stickiness is a group of proteins called mussel adhesive proteins (MAPs) which contain the catecholic amino acid 3,4-dihydroxyphenylalanine (DOPA) (Figure 1A–C) [2]. “Mussels” are the edible marine bivalves of the family Mytilidae, most of which live on exposed shores in intertidal zones, attached by means of their strong byssal threads to a hard substrate. A byssus refers to a group of strong filaments secreted by mussels when they attach themselves to hard surfaces [2]. Byssal threads, used to attach mussels to substrates, are now known as advanced bonding agents. A number of studies have shown applications of mussel adhesive or glues for industrial and surgical applications [3,4]. Additionally, byssal threads have been investigated in studies about the creation of artificial tendons [5]. Mussel adhesion is mediated by a byssus and by byssal plaques consisting of a complex array of proteins (generally mussel foot proteins, Mfps). The adhesion of Mfps to various surfaces has been widely investigated. For a better understanding of the binding mechanisms of Mfps, researchers explored the force-distance profiles and adhesion energies of three different types of Mfps, termed Mfp-1, Mfp-3, and Mfp-5, on (i) hydrophobic methyl-terminated self-assembled monolayer (CH_3_-SAM)—and (ii) hydrophilic alcohol-terminated self-assembled monolayer (OH-SAM) surfaces at various pH levels [6]. At an acidic pH, all of the Mfps samples adhered tightly to the CH_3_-SAM surfaces via hydrophobic interactions but only weakly to the OH-SAM surfaces through H-bonding. DOPA (3,4-dihydroxyphenylalanine) residues in Mfps mediate the binding to both SAM surface types, but through different interactions. The typical bidentate H-bonding by DOPA is disturbed by the longer spacing of OH-SAMs; in contrast, on CH_3_-SAMs with other nonpolar residues, it partitions to the hydrophobic surface. Asymmetry in the distribution of hydrophobic residues in proteins, the distortion of the bonding on H-bonding surfaces, and the manipulation of physisorbed binding lifetimes are important concepts in the design of adhesives and non-fouling surfaces.

**Figure 2:**
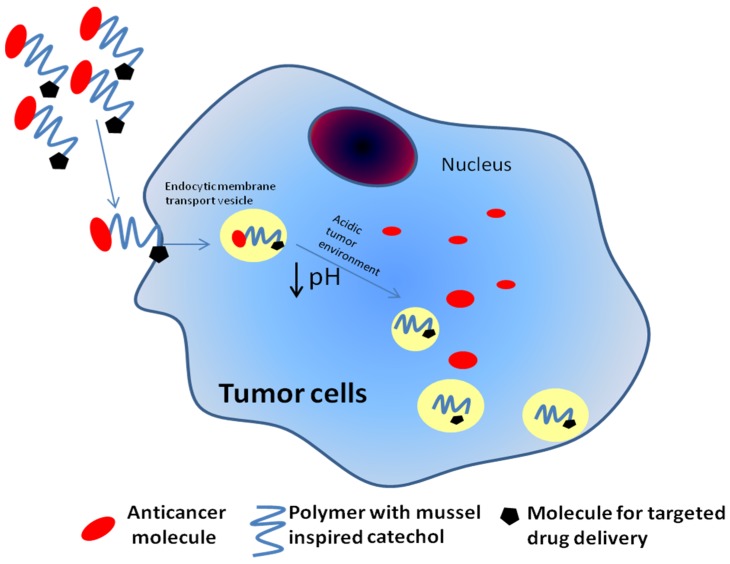
Polymers with a mussel-inspired catechol-anti-proliferative drug conjugate can be up-taken by tumor cells with the help of an endocytic cell membrane transport vesicle and can release drug molecules in the presence of the acidic environment of tumor cells.

**Figure 3:**
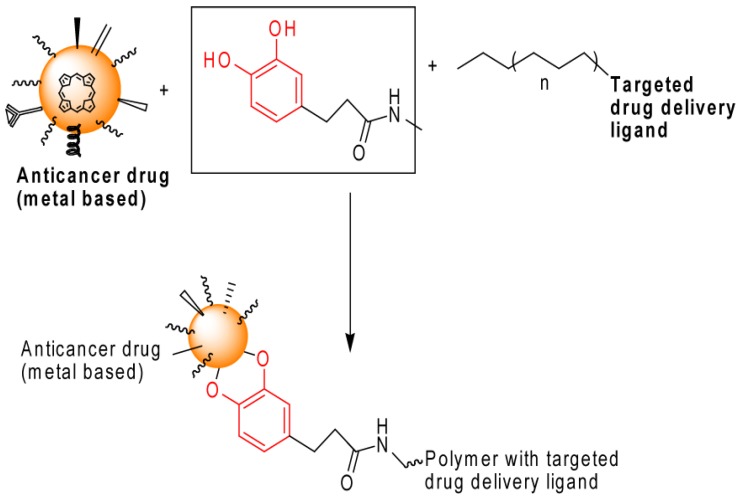
Mussel-inspired targeted drug delivery as an anticancer treatment.

Researchers have investigated the applications of mussels-inspired materials (MIMs) for the construction of ultra-small magnetic nanoparticles with catechol-derivative anchor groups which possess an irreversible binding affinity to metal oxides and thus can optimally disperse super-paramagnetic nanoparticles under physiological conditions. This not only leads to ultra-stable metal-oxide nanoparticles but also allows close control over the hydrodynamic diameter and interfacial chemistry. The latter is a crucial breakthrough in the assembly of functionalized magnetic nanoparticles [7]. This review demonstrates how an advanced and highly multifunctional material can be engineered and developed using MIMs (mussel-inspired materials). Synthetic adhesives and materials inspired by mussels have been applied extensively, and it is expected that the characterization and adaptation of many other biological adhesive strategies will follow [8]. MIMs play important roles in cancer drug delivery and in the destruction and removal of cancer cells. Recently, a mussel-inspired polymer was designed to form pH-sensitive drug delivery vehicles that are stable in the bloodstream at physiological pH levels, but become activated in acidic tumor environments, thus releasing the drug (Figure 2). A new design involves a modification of the surface of gold nanorods (NRs) with a mussel-inspired polymer coating. The NRs target cancer cells when they are irradiated, thus producing highly localized heating that destroys the cancer cells (Figure 3) [9]. Recent study integrated the chemical structure of the MAP into the design of an injectable synthetic polymer. The novel injectable citrate-based mussel-inspired bioadhesive showed controlled degradability and improved biocompatibility at a low manufacturing cost, with many more advantages in relation to current products such as fibrin glue and cyanoacrylate adhesives, which can induce allergic reactions and toxicity [10]. Injectable citrate-based mussel-inspired bioadhesives are non-toxic and are thus unlikely to cause allergic reactions and side effects. Researchers have also attempted to make the adhesion strength even stronger with no inflammatory responses [10]. In the present review, we discuss the importance of mussel bio-products and MIMs with regard to surface coatings, adhesive properties, medicine, surgery, biomedical science and bio-nanotechnology.

## 2. Applications of Mussel-Inspired Materials

### 2.1. Surface Coatings and Adhesive Applications

In the past few years, MIMs have attracted substantial interest for various types of biomedical applications, showed successful applications ranging from coatings for interfacing with cells and tissue, to drug delivery and biosensing [11,12]. Mussel adhesion is mediated by the mfps, abundant in the catecholic amino acid DOPA; however, its trend toward facile auto-oxidation is a drawback which can lead to unreliable adhesion. Yu *et* *al.* [13] revealed that mussels limit DOPA oxidation during the formation of adhesive plaque by imposing an acidic reducing system based on thiol-rich mfp, which restores DOPA by coupling the oxidation of thiols to the reduction of dopaquinone. It was also reported that the adhesive proteins secreted by mussels can be used to achieve surface reformation in a broad series of inorganic and organic materials, implying that multifunctional coatings can be fabricated for many applications [14].

**Figure 4:**
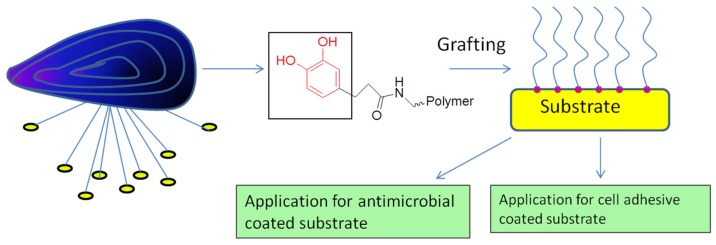
Application of mussel-inspired substrate-coated materials.

Lee *et al.* [15] introduced a simple means of surface modification in which the self-polymerization of dopamine formed an adherent polydopamine (PD) coating on a variety of materials. Coating by PD can serve as a versatile stage for secondary surface-mediated reactions, ultimately leading to metal self-assembled monolayers and grafted polymer coatings. This two-step surface modification method is distinctive in terms of its ease of application, its use of simple ingredients and reaction conditions, its applicability to many types of materials of complex shapes, and its capacity for multiple end uses, especially for antimicrobial and cell adhesive substrates (Figure 4) [15]. It has been shown that DOPA nested in hydrophobic aromatic sequences not only enhances adhesion at a neutral pH (pI or IEP) but also contributes significantly to the cohesive interactions between adhesive proteins [16]. The hydrophobic amino acid residues in the Mfp3 *slow* sequence provide DOPA with a microenvironment that retards oxidation by shielding the amino acids from the solvent, endowing the protein with the ability to maintain adhesion at a neutral to slightly basic pH. More importantly, hydrophobic interactions and inter-residue H-bonding combine to result in strong cohesion within Mfp3 *slow* layers over a relatively wide pH range [16]. This strategy provides an alternative to DOPA/metal ion chelation, and compensates in part for the limitations imposed by facile DOPA-autoxidation. By exploring the adhesive and cohesive mechanisms of bonding by the Mfp3 *slow* sequence, several studies have revealed that the wet adhesion of mussels is more complicated than a simple DOPA-mediated recipe, providing a rationale for engineering DOPA into a new generation of bio-inspired synthetic adhesive polymers. Waite *et al.* [17] reported that DOPA-containing proteins are important with regard to wet adhesion in mussels and possibly in other sessile organisms as well. Bonding depends on DOPA in both reduced and oxidized forms for adhesion and cohesion, respectively. DOPA is highly vulnerable to spontaneous oxidation, and controlling the DOPA redox is a crucial challenge when using it in adhesion applications. Mussels appear to achieve such control in their byssal attachment pad. Understanding the particulars of natural redox control may provide fundamentally important insights into adhesive polymer engineering and antifouling strategies.

**Figure 5:**
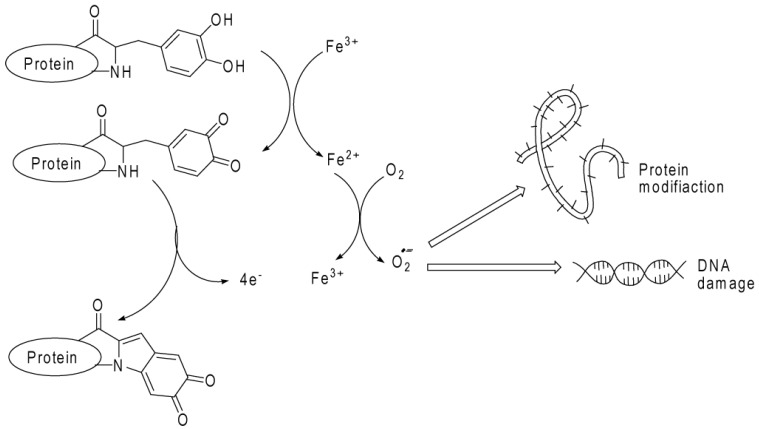
Reaction scheme presenting probable reactions of protein-bound 3,4-dihydroxy-phenylanine (DOPA). Protein-bound DOPA can be further oxidized to dopaquinone, donating two electrons to the higher valency-transition metal ions such as iron or copper present in chelates or metalloproteins. Auto-oxidation of the reduced transition metal can generate radicals such as reactive oxygen species, which can cause oxidative damage to other biomolecules. The proposed reaction of protein-bound (PB)-dopaquinone, resulting in ring closure and the release of four electrons, is described by Rodgers. Adapted the permission from [19]. Copyright © The American Chemical Society, 2000.

Researchers have investigated the composition and formation of byssal plaques and threads with the expectation of discovering technologically relevant innovations in chemistry and materials science. The DOPA residue appears to have double functionality with significant consequences for adsorption and cohesion. Nevertheless, it forms an array of weaker molecular interactions in the form of metal chelates, H-bonds, and pi-cations, which appear to dominate in terms of adsorption. On the other hand, DOPA and its redox couple, dopaquinone, can mediate the formation of covalent cross-links among byssal proteins (cohesion) [18]. Rodgers *et al.* [19] reported that protein-bound DOPA (PB-DOPA) could be formed in mammalian cells by both enzymatic pathways and radical reactions. PB-DOPA has reducing activity and the ability to cause damage to other essential biomolecules (Figure 5). The proposed reaction of PB-DOPA resulting in ring closure and the release of four electrons, was also described by Gieseg *et al.* [20]. This can be mediated through the replenishment of transition metals or from catechol-quinone-catechol redox reactions in the presence of cellular components such as ascorbate or cysteine, resulting in the amplification of radical damaging events. The formation of PB-DOPA confers on protein the capacity to chelate transition metals, generating protein “oxychelates” which may be the one factor among all factors that localize such damage. This investigation on PB-DOPA has mainly focused on detoxification and the proteolysis and excretion [17].

**Figure 6:**
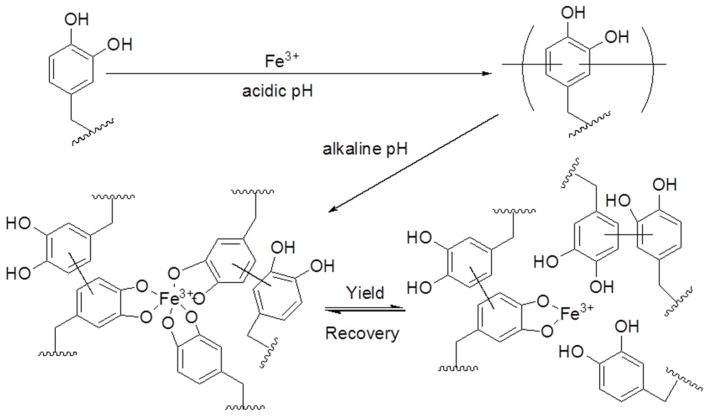
Proposed pH dependence of covalent and coordination-bond formation in catechol-polymer hydrogels containing Fe^3+^. The reaction of the catechol-terminated branched PEG with Fe^3+^ at an acidic pH results in covalently cross-linked hydrogels. Subsequent equilibration of these gels at a pH of 5, 7, or 9 introduces varying number of Fe^3+^-catechol coordination bonds that mechanically enhance the covalent network. Under the influence of a mechanical force, these coordination bonds reversibly rupture and re-form, acting as a mechanism for energy dissipation. Both oligomeric and monomeric (unreacted) catechols are believed to participate in the coordination network. Adapted with permission from [22]. Copyright © WILEY-VCH Verlag GmbH and Co. KGaA, Weinheim, 2013.

Mussel-inspired adhesive hydrogels represent novel candidates for medical sealants or glues. Brubaker and Messersmith [21] described an enzyme-degradable mussel-inspired adhesive hydrogel formulation which was achieved by incorporating the minimal-elastase substrate-peptide Ala-Ala into a branched polyethyleneglycol (PEG) structure. This system takes advantage of the neutrophil elastase expression up-regulation and secretion from neutrophils upon recruitment on wounded tissue. The degradation of the adhesive hydrogel was not observed during short-term trials involving *in vitro* treatments with elastase, though *in vivo* degradation proceeded over several months following implantation in mice. The work of Brubaker and Messersmith [21] represents the first model of an enzymatically degradable mussel-inspired adhesive and expands the potential biomedical applications of these materials. Barrett *et al.* [22] demonstrated a novel bio-inspired approach for designing extremely tough hydrogels. By regulating the pH of the reaction between catechol-terminated branched PEG and Fe^3+^, a covalently cross-linked network was prepared by the Messersmith research group with a series of coordination bonds which undertake reversible interactions to dissipate energy during the deformation process (Figure 6) [22]. Their findings show the richness of the cross-linking chemical and physical properties accessible in synthetic mussel-inspired biomaterials, which were achieved through the simple manipulation of the pH, composition, and processing method. Cautious administration of these variables provides access to a wide variety of physical properties, reflecting the stability of covalent and coordination cross-linking in the gel network. These biologically inspired hydrogels, with a viscoelastic response and water content reminiscent of hydrated natural soft tissues, represent a new set of biomaterials [22]. Previously developed synthetic polymer hydrogel tissue adhesives and sealants swell greatly under physiologic conditions, which can result in mechanical weakening and adverse medical complications. A recent report described the synthesis and characterization of mechanically hard zero- or negative-swelling mussel-inspired surgical adhesives based on catechol-modified amphiphilic poly(propylene oxide)-poly(ethylene oxide) (PPO-PEO) block copolymers [23]. Catechol oxidation at or less than room temperature resulted in a chemically cross-linked network, with subsequent warming to physiological temperatures inducing a thermal hydrophobic transition in the PPO domains and providing a mechanism for mechanical toughening. This designed approach can be easily adapted for other heat-sensitive copolymers and cross-linking strategies, representing a typical approach that can be used to manage swelling and improve the mechanical properties of hydrogels for new medical applications. Transient network hydrogels, cross-linked through histidine-divalent cation coordination bonds, were studied by Fullenkamp *et al.*, using histidine-modified star PEG polymers [24]. These biomaterials were inspired by the mussel, which utilizes histidine-metal coordination bonds to impart self-healing properties in its byssal threads. Fullenkamp *et al.* calculated pH-dependent speciation curves using equilibrium constants determined by potentiometric titration, providing insight into the pH-dependence of the histidine-metal ion coordination. It was also demonstrated that the new mussel-inspired catecholamine polymer can be used for DNA immobilization via a simple surface modification. One-step immersion of the substrate (noble metals, oxides, and polymer) in a polymer solution forms a substrate that allows the immobilization of DNA strands [25]. This method will be useful for developing DNA microarrays in various types of substrate materials with a simple preparation process. This strategy can be used for the immobilization of various types of other biomolecules, such as probes for cDNA, peptides, aptamers, or direct polymerase chain reaction (PCR) products. This method can also be used in various biomedical assays. Recently, the immobilization of trypsin on a silica and titanium support was achieved via a mussel-inspired adhesion strategy [26]. The method involves the fabrication of titanium substrates with catechol-containing biomimetic PD followed by the fabrication of trypsin on a PD layer. The immobilized enzyme maintains its catalytic activity after being coated onto PD in a wide variety of monolithic substrates.

**Figure 7:**
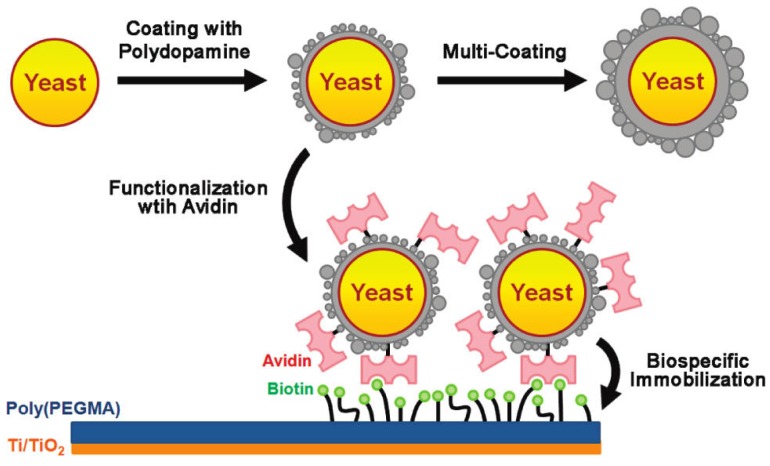
Schematic representation of the polydopamine encapsulation of individual yeast cells and the functionalization of artificial shells. Adapted with permission from [15]. Copyright © The American Chemical Society, 2011.

Recently, researchers verified the reactive encapsulation of individual yeast cells with PD, which is a biocompatible coating material inspired by the adhesive proteins of mussels [27]. This type of individual encapsulation with PD is importantly linked to the realization of artificial spores. The PD coating was found to be stable in comparison with polyelectrolyte multilayers, and effective in protecting living cells and controlling the cell division process. The PD encapsulation strategy is a good starting point for both research and for applications based on artificial spores. This strategy endows living cells with durability against harsh surroundings, provides controllability of cell cycles, and facilitates reactivity for the modification of cell-surfaces (Figure 7). The Messersmith group also introduced a two-step surface modification method in which the self-polymerization of dopamine produced an adherent PD coating on a wide range of inorganic and organic materials, including noble metals, oxides, polymers, semiconductors, and ceramics [15]. Secondary reactions can be used to create a variety of ad-layers, including self-assembled monolayers through the deposition of long-chain molecular building blocks, metal films by electroless metallization, and bioinert and bioactive surfaces via the grafting of macromolecules. In their work, Lee *et al.* [15] engineered PD surfaces for specific biomolecular interactions by forming an ad-layer of glycosaminoglycan hyaluronic acid (HA). HA/receptor interactions are important in many physiological processes, including angiogenesis, hematopoietic stem-cell commitment and homing, and tumor metastasis. A technique based on MIMs could aid in a number of surgical procedures. Among them are eyelid transplants and the correction of a ruptured fetal membrane or amniotic sac during pregnancy [28].

To create anti-coagulation and anti-hyperplasia cardiovascular devices, the polysaccharide heparin is used. An aqueous solution of DOPA and hexamethylenediamine (PDAM/HD) is used to make adhesive coatings rich in amine groups which are used for covalent heparin immobilization on stainless steel. This technique has been explored to fabricate stainless stents with heparin-retained bioactivity. Hep-PDAM/HD was also found to be a promising microenvironment for the selective enhancement of endothelial cell adhesion, proliferation, migration and the release of nitric oxide (NO), with other favorable properties as compared to control substances. These unique functions qualify the heparinized coating as an attractive alternative for the design of a new generation of stents using bio-inspired dopamine [29].

In another study [30], multifunctional mussel-inspired self-coated membranes with significant blood cell and other cell compatibilities are prepared by a green approach. Heparin-like polymer [HepLP, poly(sodium 4-vinylbenzenesulfonate)-*co*-poly(sodium methacrylate)] and heparin have been studied in relation to mussel-inspired heparin-mimicking coatings. DOPA is grafted onto HepLP or heparin to obtain DOPA-grafted HepLP (DOPA-*g*-HepLP) or DOPA grafted heparin (DOPA-*g*-Hep) using carbodiimide. A Polyethersulfone dialysis membrane is chosen as the substrate for the surface coating of the DOPA-*g*-HepLP and DOPA-*g*-Hep. The coated surface displays augmented hydrophilicity and electronegativity, decreased plasma protein adsorption, and suppressed platelet adhesion compared to a control. Heparin-coated membranes show anticoagulant bioactivities and superior performance in terms of endothelial cell proliferation and morphology differentiation. This kind of construct can have multi-biomedical applications in hemodialysis, blood purification, organ implantation, and tissue cultures systems [30].

Antifouling surfaces have been studied extensively owing to their significance in medical devices and related industries. Recently, conformal DOPA-melanin (DM) antimicrobial films were formed on various substrates by the simple method of dip-coating in a DOPA solution at a high ionic strength [31]. DM (DOPA-melanin) coatings show high hydrophilicity and the ability to bind and release cations efficiently as compared to PD. These films inhibit the growth of bacteria and demonstrate high antimicrobial activity against *S. aureus* when coated onto polycarbonate materials. Another study also investigated an antifouling system that involves the immobilization of a mussel-inspired catecholamine polypeptoid on TiO_2_ thinly coated onto a quartz surface [32]. This surface construct showed improved antifouling property as compared to other conventional antifouling surfaces such as mPEG/quartz surface. These newly developed antifouling surface constructs can be used in research areas such as single-molecule imaging, medical devices, and biosensors.

Achilles tendons (ATs) have a high rate of rupture as compared to other tendons in the human body. AT damage affects a patient’s quality of life and requires a long time for recovery. A bioadhesive coating inspired by mussel adhesion was designed and is now in use as a surgical graft material to repair AT damage. The basic mechanism for adhesion is identical to that of mussel catechols, which oxidize to quinones and are cross-linked with other catechols within the adhesive film or with functional groups such as amine and thiol biological surfaces. Polyethylene glycol and polycaprolactone are used with adhesive catechol substances due to their biocompatibility and biodegradable characteristics. The adhesive polymer was solvent-cast onto two scaffolds to determine the practicability of using the adhesive-coated concept in tendon repair. This adhesive coating demonstrated adhesive strength levels that were significantly higher than other medical adhesive materials available on the market. Furthermore, *in vivo* and clinical studies of this type of adhesive construct-augment repair will show many fruitful results in the field of biomaterials for medical applications [33].

Based on mussel-inspired reversible catechol-metal ion chemistry, a hydrogel actuator that combines ionoprinting techniques was developed [34]. Ionoprinting is based on the electrochemical oxidation of iron electrode to deposit ions onto dopamine methacrylamide (DMA) containing hydrogels. The catechol group of DMA formed a tris-complex with the deposited iron metal ion with an increase in the pH and increased cross-linking density. The difference in the cross-linking density between the ionoprinted region and the hydrogel generated enough force for the hydrogel to actuate. Hydrogel films can be changed into different three-dimentional shapes depending on the ionoprinting pattern. The actuation can be customized according to the DMA amount, applied voltage, pH and water content in the hydrogel. The capacity to form a high-stress differential while using a low concentration of catechol offers many opportunities in the design of multifunctional materials due to the focus on other functional groups in the hydrogel. This novel approach will provide a new paradigm in developing novel hydrogel actuators and will be important for the 3D printing of biomaterials for medical purposes. An inexpensive one-pot route to self-healing hydrogels with pH-tunable moduli was also presented. Recently, an advanced responsive mussel-inspired inexpensive and pH-tunable hydrogel was synthesized by reacting tannic acid, trivalent metal ions and polyallylamine in one pot [35]. This synthesized hydrogel behaves as a supramolecule below pH 8 but shows covalent strong cross-linking and can be used for various biomedical applications.

**Figure 8:**
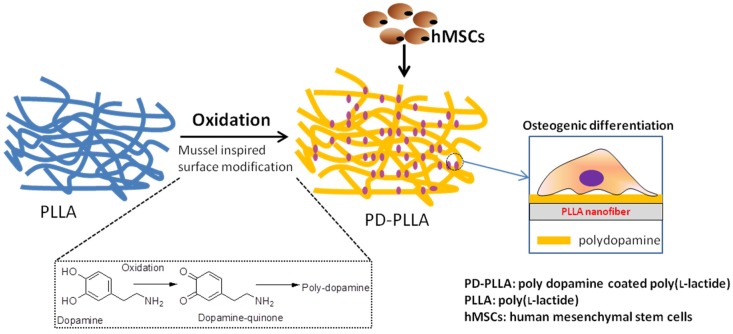
The effect of polydopamine coated Poly (l-lactide) (PLLA) fibers on the adhesion, proliferation and osteogenic differentiation of human mesenchymal stem cells (hMSCs). Adapted with permission from [36]. Copyright © Elsevier, 2012.

The development of biomaterials to direct the fate of stem cells is essential for the stem-cell-based regeneration of bone tissue. Recently, functionalized electrospun fibers using a mussel-inspired surface coating to regulate the adhesion, growth and differentiation of human mesenchymal stem cells (hMSCs) were developed (Figure 8) [36]. Poly (l-lactide) (PLLA) fibers coated with polydopamine (PD-PLLA) were prepared and incubated in a dopamine solution for 1 h, resulting in the formation of PD with less of an effect on the roughness and hydrophobicity of the fibers. The prepared PD-PLLA fibers modulated the hMSC responses in many ways. Importantly, the adhesion and proliferation of hMSCs cultured on PD-PLLA were significantly enhanced relative to those cultured on PLLA alone. In addition, hMSCs cultured on PD-PLLA demonstrated the up-regulation of genes associated with osteogenic differentiation as well as angiogenesis. These outcomes indicate that the simple bio-inspired surface modification of organic fiber substrates using PD is a very promising means of regulating stem-cell functions, possibly allowing the realization of effective stem-cell delivery carriers for bone tissue engineering applications [36].

### 2.2. Application of Mussel-Inspired Materials in Medicine and Surgery

The marine microenvironment and the human body have many similarities. Understanding the physics and chemistry of marine bio-products will provide scientists with insight into the design of biomaterials for medical applications.

The conversion of tyrosine to DOPA is an important event in the processing of the adhesive protein in mussels as there are multiple roles played by DOPA at the interfaces in the adhesion process. For inorganic surfaces, unoxidized DOPA forms high-strength but reversible coordination bonds; however, on organic surfaces, it is able to adhere to surfaces via the formation of covalent bonds. This remarkable ability to adhere to both organic and inorganic surfaces is related in part to the equilibrium that exists between DOPA and dopaquinone at marine pH levels, allowing the species to interact with surfaces. It is also notable that the strong bonds between DOPA and organic and inorganic surfaces are formed in the presence of sea water, which is likely a fundamental feature of protein-based glues which operate in wet oceanic environments. The use of DOPA and related catecholic molecules has recently been considered as part of a potential means of anchoring macromolecules onto oxide surfaces for medical applications [37]. Researchers found that adhesive proteins secreted by mussels serve as a stimulus behind the versatile approach to the surface modification of a wide range of inorganic and organic materials [14]. Recent work has also provided an overview of the recent developments in PD-based materials, including the synthesis of nanoparticles, capsules, and structural mechanisms as well as their physicochemical and biomedical properties [38]. Researchers have synthesized polymers containing DOPA in an effort to create water-resistant adhesives with self-healing and biocompatible properties [39].

**Figure 9:**
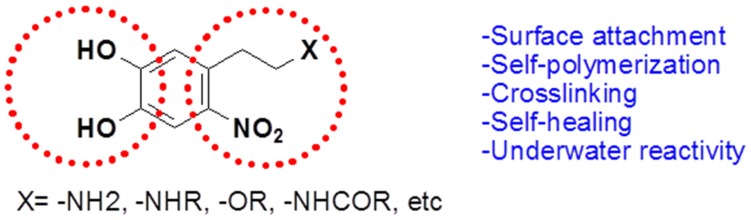
Structure of nitro-dopamine derivatives.

Scientists at the Max Planck Institute for Polymer Research in Mainz, Germany, led by Aránzazu del Campo, synthesized an underwater adhesive that bonds strongly to various types of surfaces [39]. In contrast to previous mussel-inspired adhesives, this new glue is reversible, as it degrades and detaches when exposed to light. It is also biocompatible and can repair itself, fusing on its own when cut. This adhesive material can be used for closing wounds and in drug delivery patches, detachable scaffolds for tissue regeneration, and substrates for cell engineering. Campo *et al.* [39] create their waterproof glue with nitrodopamine, a natural molecule that is related to DOPA. They reported that the adhesive properties of nitrodopamine are more stable than those of DOPA (Figure 9). This allows the cross-linking and solidification of the adhesive in a manner similar to the process used by the mussels, but the adhesive degrades when it is exposed to light of a particular wavelength. This synthesized nitrodopamine are identical to the adhesives of mussels, but they can be detached on demand.

Recently, Bilic *et al.* [40] investigated injectable surgical sealants that are biocompatible with fetal membranes, with the potential to be utilized for the closure of iatrogenic membrane defects. They used dermabond^®^, Histoacryl^®^, and Tissucol fibrin glues along with three types of *in situ* formed PEG-based polymer hydrogels in a bonding and acute toxicity study upon direct contact with fetal membranes. They found that biological Tissucol fibrin glue has appealing properties for membrane sealing as compared to other glues. They also developed a new synthetic hydrogel formulation in the form of a mussel-mimetic sealant and the biologic Tissucol fibrin glue with good properties for membrane sealing. Extracts of all adhesives were shown to be non-toxic for cultured cells. Tissucol and one PEG-based hydrogel, which is a type of mussel-mimetic tissue glue, demonstrated efficient, non-disruptive, non-toxic bonding to fetal membranes. Their synthetic hydrogel-type tissue adhesive, which merits further evaluation *in vivo,* has emerged as a potential sealing modality for iatrogenic membrane defects. These results demonstrated that mussels glue efficiently seals elastomeric membranes under wet or moist conditions with comparable viscoelastic properties [41]. *Ex vivo* studies also showed that proteolytic degradation does not affect mussels or mussel-inspired glues [42]. These materials display good stability in proteolytic environments, which makes them a favorable sealing material for future applications.

**Figure 10:**
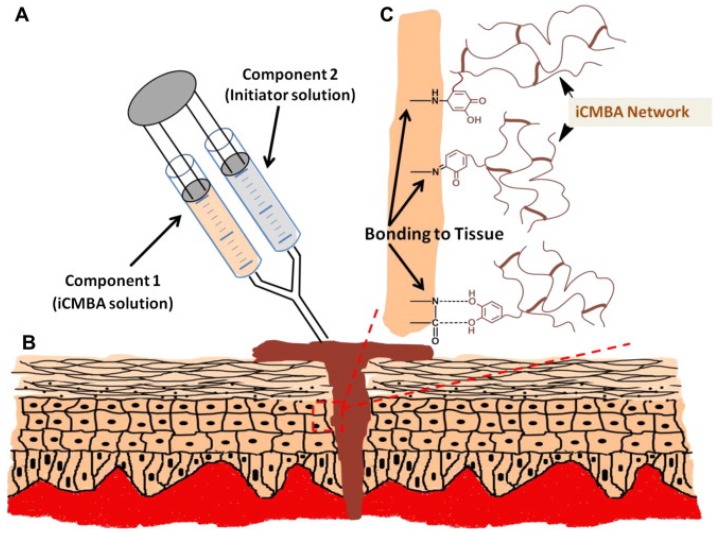
Representation of the injectable citrate-based mussel-inspired bioadhesives (iCMBA) application for wound closure: (**A**) Preparation and application of a two-component adhesive consisting of iCMBA and oxidizing (sodium periodate) solutions; (**B**) Schematic representation of iCMBA utilized for sutureless wound closure; (**C**) Proposed mechanisms of iCMBA adhesion to tissues. Reprinted with permission from [10], Copyright © Elsevier, 2012.

**Figure 11:**
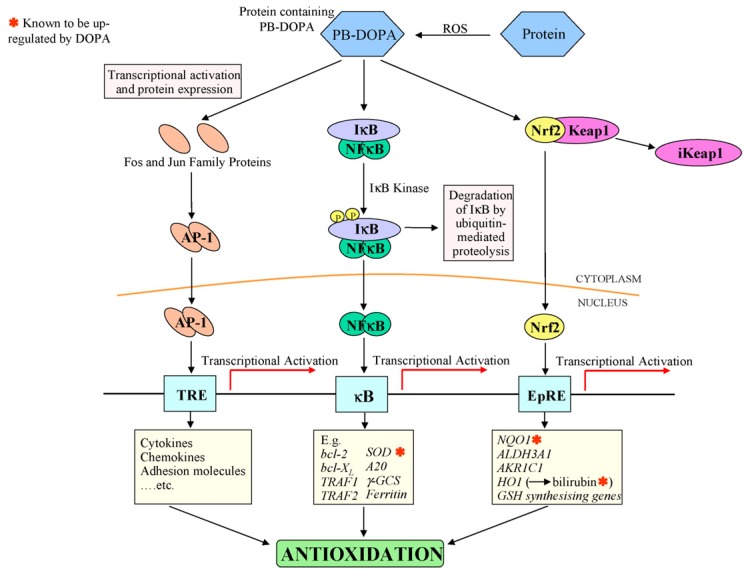
Scheme illustrating the hypothetical induction of antioxidant defenses by PB-DOPA. Reproduced with permission from [45]. Copyright © Elsevier, 2007.

Yang *et al.* [10] recently developed injectable citrate-based mussel-inspired bioadhesives in the form of a biodegradable strong wet-tissue adhesive that can effectively close a bleeding wound, thus stopping the bleeding and helping with tissue regeneration without the aid of surgical tools. The injectable citrate-based mussel-inspired bioadhesives can be used for topical and non-topical applications across all disciplines of surgical practice, ranging from a suture/staple replacement; tissue grafts to treat hernias, ulcers, and burns; hemostatic wound dressings for laparoscopic partial nephrectomy; waterproof sealants for vascular anastomoses; and for the treatment of gastrointestinal fistulas, leaks, mucosal oozing or bleeding, and perforations (Figure 10) [10]. Inspired by a mussels byssus secretion through a pH jump, Holten-Andersen *et al.* [43] developed a simple method to control catechol-Fe^3+^ interpolymer cross-linking via changing the pH. The Raman resonance signature of catechol-Fe^3+^ cross-linked polymer gels at high pH levels is similar to that of natural adhesives secreted by mussels. These gels display elastic moduli (G′) that approach those of covalently cross-linked gels as well as self-healing properties [43]. There is an urgent need for medical adhesives that function reliably on wet tissue surfaces with minimal inflammatory responses. To address these performance characteristics, Brubaker *et al.* [44] generated a synthetic adhesive material inspired by mussels. The *in vivo* performance of the material was assessed in a mouse extra-hepatic syngeneic islet transplantation model. They designed the adhesive polymer with a branched PEG core whose end groups were derivatized with catechol, a functional group inspired by mussels adhesive proteins. In an oxidizing environment, the adhesive forms within a minute from catechol-derivatized PEG (cPEG) solutions. The cPEG adhesive elicited minimal inflammatory responses in mice and maintained a bond with supporting tissue for up to one year upon implantation. The synthesized cPEG adhesive was shown to immobilize transplanted islets at epididymal fat pads and external liver surfaces efficiently, permitting normoglycemic recovery and graft revascularization. The findings by Messersmith *et al.* [44] established the use of mussel-inspired adhesives for islet transplantation. Nelson *et al.* [45] developed protein hydroperoxides and protein-bound 3,4-dihydroxyphenylalinine as the key redox-active products during free radical attacks on proteins. Protein-bound 3,4-dihydroxyphenylalinine forms a redox cycle between the catechol and quinone forms and binds transition metals, while hydroperoxides are converted to stable hydroxides. The free amino acid 3,4-dihydroxy phenylalinine, an oxidation product of tyrosine, is a normal metabolite which is involved in the pathways of dopamine and melanin production. On the other hand, the physiological levels of protein-bound 3,4-dihydroxy-phenylalinine are very low, though remarkably elevated levels occur in some pathologic conditions. Unlike free 3,4-dihydroxyphenylalinine, protein-bound 3,4-dihydroxyphenylalinine has been proposed as a signal for the activation of cellular defenses against both oxidative fluxes during oxidative stress and oxidative damage, which occasionally develop. For distinctly free 3,4-dihydroxyphenylalinine, the levels of protein-bound 3,4-dihydroxyphenylalinine can change by five to ten times during oxidative damage *in vivo*, which can serve as an appropriate property for signaling molecules. Several mechanisms by which protein-bound 3,4-dihydroxyphenylalinine may trigger oxidative defenses via NF-κB and other transcription factors have been suggested (Figure 11). Many effects of 3,4-dihydroxyphenylalinine in these situations may be mediated by the production and actions of protein-bound 3,4-dihydroxyphenylalinine [45].

**Figure 12:**
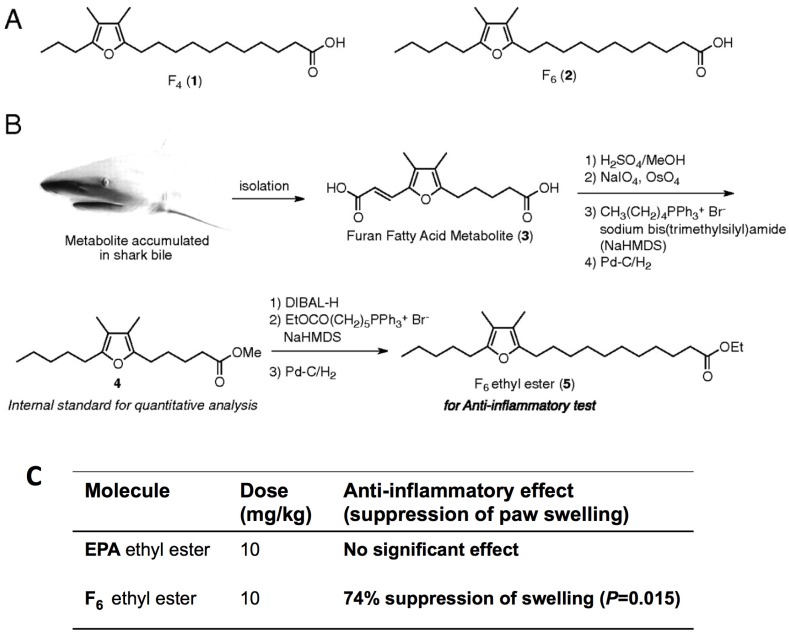
(**A**) Structures of typical F-acids detected in the green-lipped mussels; (**B**) Partial synthesis of furan fatty acid F6, and (**C**) Inhibition of adjuvant-induced arthritis in Wistar rats. The ordinate is the increase in the volume of the rear left paw between the beginning (day 10) and the end of the dosing (day 15) as described by Wakimoto *et al.* [46]. Reprinted with permission from [46]. Copyright © PNAS, 2011.

Wakimoto *et al.* [46] demonstrated that furan fatty acids (F-acids) are a minor component of the fatty acids in the lipid extract of the New Zealand green-lipped mussels and partially synthesize furan-6 fatty acid and display more potent anti-inflammatory activities than eicosapentaenoic acid (EPA) (Figure 12). Their study sheds light on F-acids as potential anti-inflammatory agents and paves the way for more thorough examinations of the anti-inflammatory efficacy of the New Zealand green-lipped mussels. They noted that the New Zealand green-lipped mussels may be a preferred food for the efficient intake of F-acids and posited that they can be consumed raw or as stabilized oil extract. Li *et al.* [47] also reported that hard-shelled mussels lipid extract (HMLE) at a dose of 100 mg/kg of body weight possesses similarly strong anti-inflammatory activity compared to New Zealand green-lipped mussels lipid extract (GMLE), diminishing hind paw swelling and arthritis indexes and improving body weight gain in both adjuvant-induced (AIA) and collagen-induced arthritis (CIA) in rats. This strong efficacy may be associated with the down-regulation of inflammatory mediators (LTB4, PGE2, TXB2), pro-inflammatory cytokine (IL-1β, IL-6, IFN-γ, TNF-α) production, MMP (MMP1, MMP13) mRNA expression, and the up-regulation of anti-inflammatory cytokine (IL-4, IL-10) production and TIMP1 mRNA expression in the serum and joint tissues of arthritic rats. They also found no hepatotoxicity in AIA rats that received HMLE and GMLE [47]. Beaulieu *et al.* [48] reported anti-proliferative activities in blue mussels (*Mytilus edulis*) by-products. They tested fractions on four cancerous cell lines: A549 lung adenocarcinoma, BT549 breast tumor, HCT15 human colon tumor and PC3 prostrate cancer. The 50 kDa fraction, enriched in peptides, presented anti-proliferative activity in all cell lines. They claimed that hydrolysates formed due to fractionation from *Mytilus edulis* after enzymatic hydrolysis. At a protein concentration of 44 µg/mL, the 50 kDa fraction induced mortality rates of 90% for PC3, 89% for A549, 85% for HCT15 and of 81% for the BT549 cell lines. The 50 kDa fraction consists of 56% of proteins, 3% of lipids and 6% of minerals on a dry weight basis and the lowest levels detected of taurine and methionine and the highest levels of threonine, proline and glycine amino acids. Their study suggests that *Mytilus edulis* by-products should be viewed as high-value products with strong potential as anti-proliferative agents.

Polymeric materials that basically heal at damage sites under wet conditions are urgently needed for biomedical applications. Recent research has demonstrated self-repairing in the metal-free water of synthetic polyacrylate and polymethacrylate materials that are surface-modified with mussel-inspired catechols [49]. This construct initiated by H-bonding between catechol functional groups supports other deep physical non-covalent interactions contributed by subsurface moieties. These self-mending properties of materials can be applied in the medical field in the design of more durable implants in the future.

A mussel-inspired surface functionalization to immobilize bioactive peptides for the enhanced endothelialization of decellularized vein matrixes (DVMs) was developed by Lee *et al.* [50]. These authors were able to transform DVMs with extracellular matrix-derived cell-adhesion peptides by mean of PD coatings. PD-mediated peptide immobilization enhanced the adhesion, metabolic activity, and endothelial differentiation of human endothelial progenitor cells. Their strategy for facilitating enhanced endothelialization would be useful for improving the function of small-diameter tissue-engineered blood vessels which can be used in cases of thrombosis, intimal hyperplasia, and calcification. Decellularized matrices engineered with PD-peptide modifications will be a great invention after further evaluations through long-term cultures of endothelial progenitor cells and by *in vivo* trials. Moreover, Qin and Buehler [3] reported that mussels could help to create artificial tendons. Through a combination of experimentation and a simulation, they demonstrated that the heterogeneous material distribution in byssus threads plays an important role in decreasing the effect of impact loading. They found that a combination of stiff and soft materials at a ratio of 80:20 enables mussels to dissipate impact energy rapidly and effectively. Notably, this facilitates significantly enhanced power under dynamical loading that exceeds 900% of the strength under static loading.

### 2.3. Application of Mussel-Inspired Materials in Biomedical Nanotechnology

The biological activity of nanoparticles is strongly dependent on the surface properties of the substrate. The present part illustrates the use of mussel-inspired proteins in the fabrication of functionalized bio-inspired nanomaterials leading to the possible realization of important biomedical applications.

Amstad *et al.* [7] developed catechol-derivative anchor groups which possess irreversible binding affinity to iron oxide and can thus optimally disperse super-paramagnetic nanoparticles in a physiologic environment this strategy can provide ultra-stable iron oxide nanoparticles, especially under medically relevant conditions of elevated temperatures and ionic strength levels. It can also control the hydrodynamic diameter and interfacial chemistry. Their work was an important breakthrough in the assembly of functionalized magnetic nanoparticles, e.g., as targeted magnetic resonance-contrast agents. This ideal nanoscale drug delivery vehicle allows control over space-time-released doses. This is achieved by stealth liposomes consisting of self-assembled super-paramagnetic iron-oxide nanoparticles which are individually stabilized with palmityl-nitro DOPA incorporated into the lipid membrane [51]. Alternating magnetic fields were used to control the timing and dose of repeatedly released loads from such vesicles by local heating of the membrane, which changed its permeability without major effects on the environment. Recently, researchers deposited mussel-inspired PD onto gold nanorods, after which antibodies were able to bind to PD-coated nanorods. Anti-epidermal growth factor receptor (EGFR)-PD were stable in media and were specifically bound to EGFR-overexpressing cells. Illumination of the cells targeted with anti-EGFR-PD nanorods enhanced cell death compared to non-irradiated controls and cells treated with antibody-free nanorods. PD facilitated the surface functionalization of gold nanorods with biomolecules, allowing cell targeting and the photo-thermal killing of cancer cells. PD can potentially coat a large variety of nanostructures with targeting ligands as a strategy for the bio-marine environment [52]. Researchers utilized silver nitrate to oxidize the polymer catechol, leading to covalent cross-linking and hydrogel formation with an immediate reduction and release of Ag (I) [52]. The hydrogels were found to inhibit microbial growth, consistent with the well-known antibacterial properties of silver, while having less toxic effects on mammalian cell viability. One report explains a novel procedure which can be used to immobilize magnetic particles onto whole the *Gluconobacter oxydans* via a synthetic adhesive biomimetic material inspired by the protein adhesive of mussels [53]. Their approach involves the simple coating of a cell-adherent PD film onto magnetic nanoparticles followed by the conjugation of PD-coated nanoparticles to *G. oxydans* and resulting in cell aggregation. Importantly, the G. oxydan aggregates showed high specific activity and good reusability. This facile approach offers potential advantages of a low cost, easy cell separation, low diffusion resistance, and high efficiency. Moreover, this approach is a convenient platform technique for the magnetization of cells *in situ* via direct mixing of nanoparticles with a cell suspension. Another study demonstrated that air-plasma-treated electrospun PCL and PLA nanofibers can be used as carriers for the loading and release of charged molecules in a pH-responsive manner [54]. It also demonstrated that a mussel-inspired protein PD coating could finely tailor the pH-responsive loading kinetics and release of charged molecules. These new formulations may have prospective applications in drug delivery to specific targets that are related to variations in the pH level [54].

Recently gold@silver core-shell NRs were prepared using a novel biomimetic method, including deposition of a thin organic PD primer onto gold NRs surfaces, followed by spontaneous electroless silver metallization and the conjugation of antibacterial antibodies and passivating polymers for targeting to Gram-negative and Gram-positive bacteria. These nanorods showed high cytotoxicity on *S. epidermidis* and *E. coli* cells upon exposure to light due to the combined antibacterial effects of plasmonic heating and the release of silver [55]. In another study inspired by biomimetic PD, researchers used PD as a coating of photoluminescent graphene quantum dots (GQDs) through a simple exfoliation and oxidation process to increase the level of stability and efficiency [56]. The PD-coated GQDs show better photoluminescent stability levels than non-coated GQDs, high stability for long time, and can be used as a single-cell imaging agent and for drug and gene delivery [56]. PD-functionalized graphene oxide (GO) also showed increased compatibility compared to unmodified GO on blood cells [57]. GO can be used for many biomedical applications, such as drug delivery, imaging, and photothermal therapy. However, hemolysis in blood cells occurs due to GO amphiphilicity and surfactant-like behavior. In the case of PD, modified GO interacts with the membrane lipid bilayer of blood cells and significantly suppresses toxicity, which also makes it possible to use modified GO for various important biomedical applications. PD-coated carbon materials also show increased compatibility with human cells. The increased compatibility is related to the increased water solubility of carbon nanomaterials, which provides a more suitable environment for cell growth than uncoated carbon materials [58,59].

Recently, chemically cross-linked fracture-resistant nanocomposite hydrogels were prepared using dopamine methacrylamide consisting of a biomimetic side chain, inorganic nano-silicates, and Laponite [60]. The prepared nanocomposite hydrogels showed improved stiffness as well as exceptional energy dissipation capabilities. Nanocomposite hydrogels showed full compressive stress, elastic modulus, toughness, and storage and loss moduli values that were higher than control gels. The catechol side chain of dopamine methacrylamide possibly formed strong physical bonds with Laponite, which can dissipate the fracture energy while minimizing permanent damage to the network architecture and can be used for various biomedical and medical applications.

A human gelatin nano-coating was developed by conjugating dopamine with carbodiimide, the surface modifier, to form bio-adhesive surfaces [61]. Human umbilical endothelial cells were used to check cell attachment and growth on the modified surfaces. The binding strength is significantly enhanced by the recombinant gelatin through the conjugation with dopamine onto a titanium surface. Human umbilical endothelial cell attachment is also enhanced on dopamine conjugated gelatin nano-constructs. This dopamine-conjugated gelatin nanocoated surface is useful for many biomedical purposes, such as a human cell culture system and tissue engineering applications related to the capture and sequestering of growth factors to support cell proliferation.

Enhanced cell adhesion and migration were shown by newly construct monomeric catechols on the surfaces of polymeric nanofibers. The nanofiber (NF) of poly(ε-caprolactone)-polyethylene glycol-amine, fabricated with dihydroxyphenyl propionic acid, displayed catechol moieties on the surface [62]. Catechol on the NFs showed high cell viability, enhanced cell adhesion and good migration as compared to control NFs. These newly developed catecholized NFs can be used in tissue engineering scaffolds with enhanced cell adhesion and migration properties by adding a small amount of catechol. In another study, poly (ε-caprolactone) NFs fabricated with mussel-inspired DM were used for the neuronal differentiation of hMSCs [63]. A mussel-inspired DM coating used for the adsorption of RE-1 silencing transcription factor (REST) onto the scaffold. The mussel-inspired scaffold-mediated sustained release of siRNA promoted the differentiation of stem cells by targeting REST. This construct showed high loading efficiency and reduced burst release of siRNA. DM modification and fiber alignment further enhanced the siRNA loading, release kinetics and REST knockdown efficiency. These newly fabricated scaffolds have many potential applications, such as enhancing MSC neuronal differentiation in non-specific environments. Recently, recombinant mussel adhesive protein (rfp-1), Fe(III)-DOPA complexes and polycaprolactone were used to fabricate mussel-inspired electrospun NFs for applications related to tissue engineering scaffolds [64]. These fabricated NFs are based on recombinant mussel-associated protein rfp-1 and its Fe(III)-DOPA complexation chemistry and their mechanical properties can be modulated by varying the pH.

A three-dimensional (3D), ultralight, mussel-inspired dopamine-fabricated nitrogen-doped graphene aerogel was also developed [65]. Dopamine was self-polymerized on graphene surfaces and embeds nitrogen atoms on pyrolysis. The aerosol showed outstanding electrochemical activity towards the oxidation of biomolecules such as ascorbic acid, dopamine, and uric acid, playing important roles in the physiological functioning of organisms in neutral media based on the 3D open-pore structure and nitrogen doping. The fabricated nitrogen doped graphene aerogel is mechanically stable and fire-resistant and can be used for biomedical applications in the future.

Gold nanorods, fabricated with biomimetic PD, have been recently used for exposing antibodies to target tumor cells as a photothermal therapy [66]. PD was polymerized onto nanorods, and epidermal growth factor receptor antibodies (anti-EGFR) were immobilized on the construct. The anti-EGFR-PD-nanorods specifically and efficiently bind to EGFR-overexpressing cancer cells and show significant anticancer effects compared to negative and positive controls. Dopamine-fabricated magnetic iron oxide nanoparticles are also used recently for the immobilization of lipase proteins [67]. This conjugated magnetic nanoparticle showed a high enzyme loading capacity and strong adhesive interaction between lipase and PD. Enzyme maintained its enhanced stability and activity as compared to control free lipases under various conditions. This new, efficient, facile and economical strategy for immobilization of enzymes demonstrate high rate of reusability and recovery. Single-nanoparticle@metal-organic framework (MOF) core-shell nanohybrids were constructed using strategy based on mussel-inspired PD [68]. Specifically, nanohybrids with gold nanoparticles sandwiched between the magnetic core and MOF shell through localized reduction by mussel-inspired PD demonstrating flexible, rational functionality integration can be used in the field of nanomedicine. These core-shell nanohybrids also have molecular sieving properties and can be used for controlled drug release, sensing and catalytic applications.

Fe_3_O_4_@polydopamine nanoparticles are also used in a pH-sensitive manner for the controlled drug release of anticancer drugs (e.g., bortezomib) via reversible bonding between the catechol and boronic acid groups of PD and anticancer drugs [69]. The sustained release of the anticancer drug at a greater concentration was achieved at a lower pH. These core-shell Fe_3_O_4_ PD nanoparticles can serve as a platform for responsive drug delivery, a recyclable catalyst support and an adsorbent. These constructs of PD onto magnetic nanoparticles have unique features which allow them to act as a versatile platform for multiple biomedical applications. For drug delivery, polymer capsules are also considered to be desirable transport vehicles. A monodisperse PD capsule is prepared by the one-step interfacial polymerization of dopamine on dimethyldiethoxysilane emulsion droplets [70]. The size of the cargo-loaded PD capsule is in the range of 400 nm to 2.4 µm, and it can be tailored by changing the emulsion concentration used with the template or the emulsion condensation time. Stabilized magnetic (Fe_3_O_4_) nanoparticles, fluorescent quantum dots, and anti-cancer hydrophobic drugs can be preloaded in the emulsion droplets, and these advanced functional materials remain encapsulated in the capsules. This strategy provides a new avenue for PD polymer capsule preparation and encapsulation, and can be used in many biomedical applications. Recently for developing highly efficient nanoconjugates, Fe_2_O_3_ nanoparticles are functionalized also using an approach involving mussel-inspired dopamine with human serum albumin for the labeling of various types of cell lines, including stem cells [71]. These prepared nano-constructs are nontoxic and can be used for the efficient *in vivo* magnetic resonance imaging with xenograft and focal cerebral ischemia models.

## 3. Conclusions

DOPA-containing proteins are crucial for wet adhesion in mussels in a microenvironment that retards oxidation by shielding the amino acids from the solvent and endowing the protein with the ability to maintain adhesion at neutral to slightly basic pH levels. More importantly, hydrophobic interactions and inter-residue H-bonding combine to result in strong cohesion within Mfp3 layers over a relatively wide range of pH levels. By exploring the adhesive and cohesive mechanisms of bonding by the adhesive proteins of mussels, recent studies reveal that the adhesion of mussels is more complicated than a simple DOPA-mediated procedure, thus providing the motivation for a new generation of bio-inspired synthetic adhesive polymer materials. Some findings also explained the roles of hydrophobic and hydrophilic interactions in both biological and non-biological instances of mussels and mussel-inspired adhesion. The issues of redox and adhesion may be much broader and not limited solely to mussel DOPA chemistry. Understanding the aspects of natural redox control can provide fundamentally important insight for adhesive polymer engineering, surface modification and antifouling strategies.

Mussel-inspired adhesive exhibit attractive properties for use immediately following tissue injuries, for applications in soft tissues including the gut and the vasculature, and for adhesion in an inflamed environment (*i.e.*, hernia). Research on mussel-inspired medical adhesives has also focused on tissue-specific medical applications, including wound care for diabetic patients, sutures for corneal tissue, and the surgical repair of nerves. MIMs are also capable of repairing defects in human fetal membranes, and they are biocompatible and effective at sealing small holes. The mussels unstable fatty acid components may have potent anti-inflammatory effects, which will be important for various types of autoimmune and inflammatory diseases. Some fractions of peptides from mussels show significant anti-proliferative activities with high nutritive potential as a source of protein, vitamin C, iron, zinc and omega-3. Mussel-inspired controlled drug delivery systems are also a promising emerging area for research. Some advanced types of novel formulations or conjugations with nanomaterials can also lead to the realization of applications in drug delivery to specific targets that are related to variations in pH levels and other physiochemical factors. Using a biodegradable mussel-inspired polymer surface coating also can regulate the adhesion, proliferation and differentiation of human mesenchymal stem cells. Collectively, we can conclude here that MIMs are important for many types of biomedical applications and that many more without immunogenicity issues will be explored by scientific communities and industries in the future.
